# Supplementary material for: Conformational ensemble of the human TRPV3 ion channel
Source: Nat Commun. 2018 Nov 14;9:4773. doi: 10.1038/s41467-018-07117-w (PMC6235889; doi:10.1038/s41467-018-07117-w)
Supplement: Supplementary file 1 — Supplementary Information [file 41467_2018_7117_MOESM1_ESM.pdf]

Supplementary Information

Conformational ensemble of the human TRPV3 ion channel

Zubcevic et al

10 Supplementary Figures

1 Supplementary Table

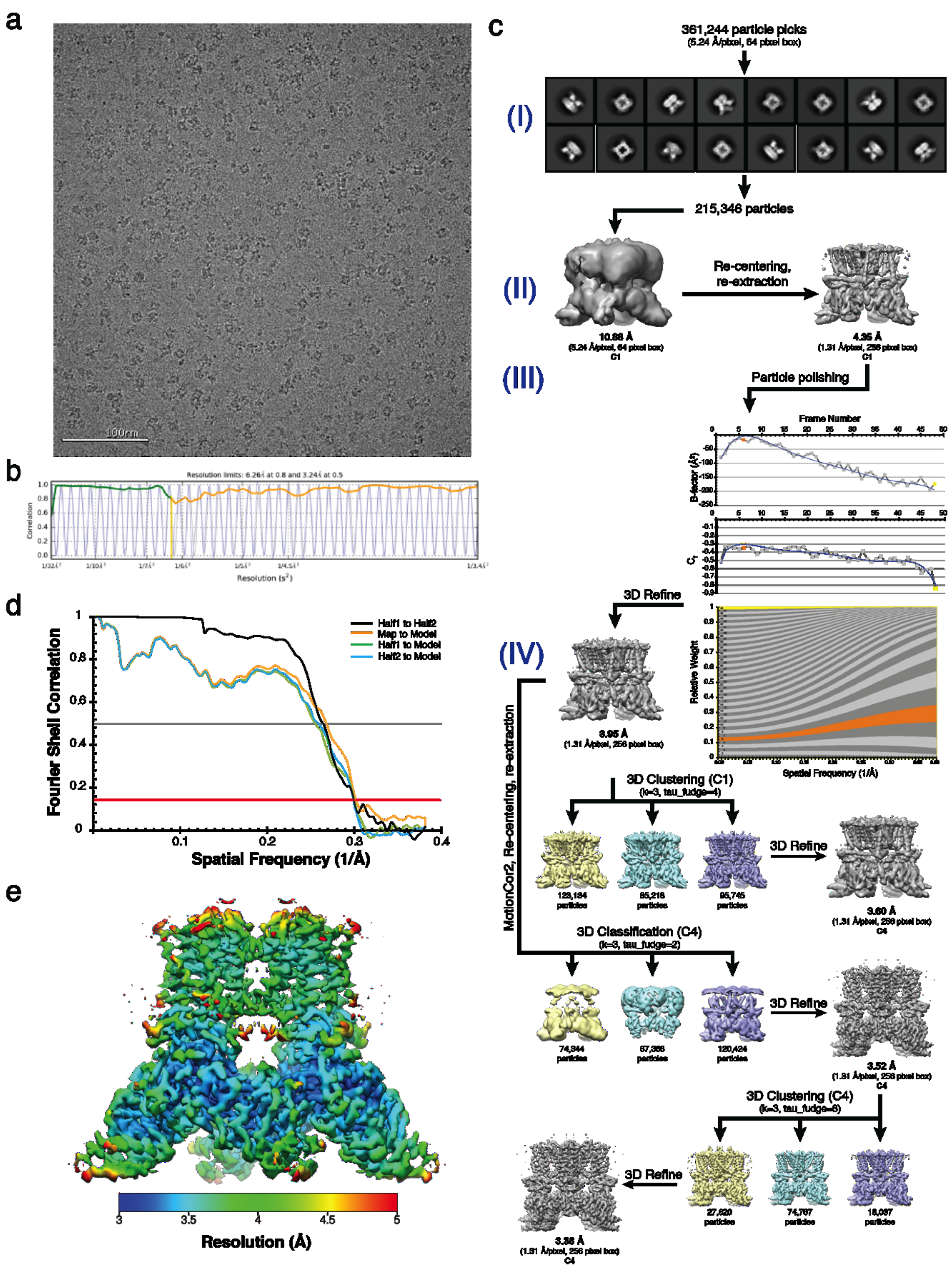

Supplementary Fig. 1 Cryo-EM data collection and processing for the apo TRPV3 channel a, Representative micrograph of apo TRPV3 in vitreous ice. **b,** CTF estimation fit (shown in blue) to experimental data and colored by CC (green, CC ≥90). **c,** Particles were

extracted from aligned micrographs and subjected to **(I)** reference-free 2D classification using RELION. Representative 2D class averages are shown. Particles comprising the "best" class averages were **(II)** 3D auto-refined and subjected to **(III)** RELION particle polishing, followed by **(IV)** two rounds of 3D classification. The number of contributing particles are listed below each class. A final subset of 27,620 particles exhibiting C4 symmetry was auto-refined to yield a final reconstruction at ~3.4 Å resolution. **d,** FSC curves calculated between the half maps (black line), atomic model and the final map (orange line), and between the model and each half-map (green and blue lines). **e,** Local resolution estimate of the final reconstruction calculated using BSOFT.

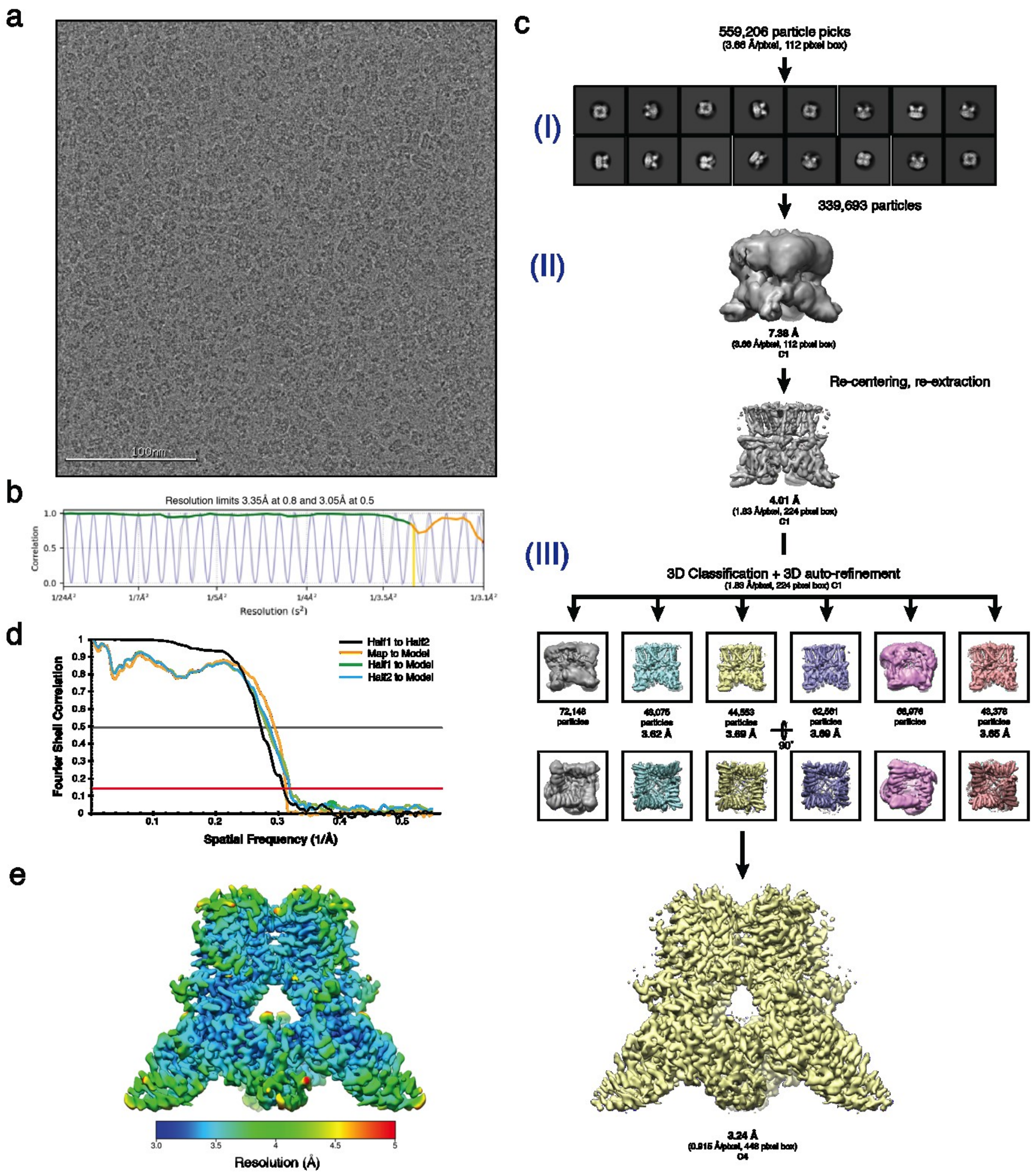

Supplementary Fig. 2 Cryo-EM data collection and processing for the sensitized TRPV3 channel **a,** Representative micrograph of sensitized TRPV3 in vitreous ice. **b,** CTF estimation fit (shown in blue) to experimental data and colored by CC (green, CC ≥90). **c,** Particles were extracted from aligned micrographs and subjected to **(I)** reference-free 2D classification using RELION. Representative 2D class averages are shown. Particles

comprising the "best" class averages were **(II)** 3D auto-refined and subjected to **(III)** 3D classification. The number of contributing particles are listed below each class. A final subset of 44.553 particles exhibiting C4 symmetry was auto-refined to yield a final reconstruction at ~3.2 Å resolution. **d,** FSC curves calculated between the half maps (black line), atomic model and the final map (orange line), and between the model and each half-map (green and blue lines). **e,** Local resolution estimate of the final reconstruction calculated using BSOFT.

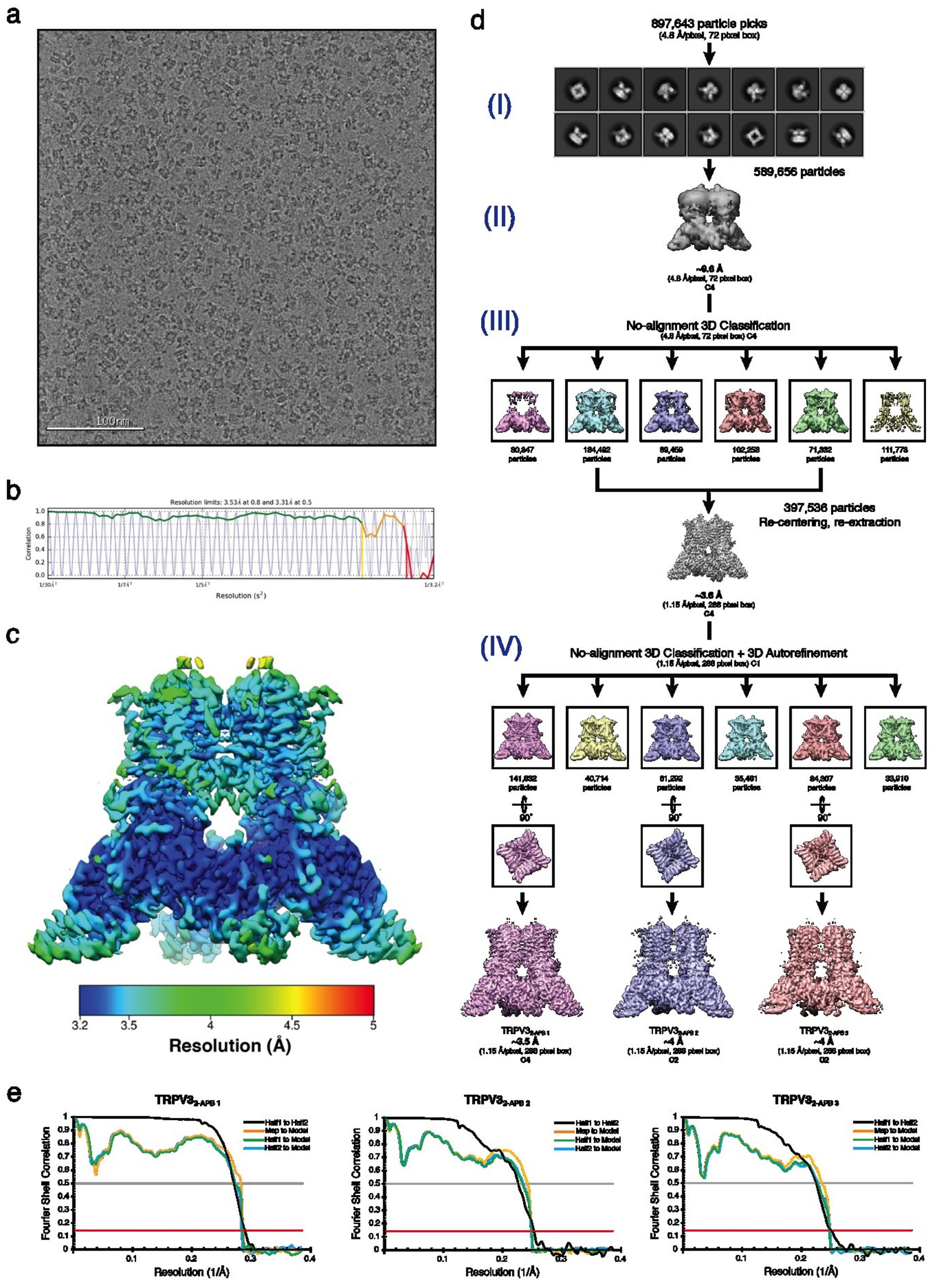

Supplementary Fig. 3 Cryo-EM data collection and processing for the TRPV3 channel in the presence of 2-APB a, Representative micrograph of TRPV3 in the presence of 2-APB

in vitreous ice. **b,** CTF estimation fit (shown in blue) to experimental data and colored by CC (green, CC ≥90). **c,** Local resolution estimate of the final $TRPV3_{2\text{-}APB\ 1}$ reconstruction calculated using BSOFT. **d,** Particles were extracted from aligned micrographs and subjected to **(I)** reference-free 2D classification using RELION. Representative 2D class averages are shown. Particles comprising the "best" class averages were **(II)** 3D auto-refined and subjected to **(III)** 3D classification without alignment with C4 symmetry imposed, followed by **(IV)** an additional round of no-alignment asymmetric 3D classification. The number of contributing particles are listed below each class. One state, exhibiting C4 symmetry, was auto-refined to ~3.5 Å ($TRPV3_{2\text{-}APB\ 1}$), and two distinct states exhibiting C2 symmetry both refined to ~4 Å ($TRPV3_{2\text{-}APB\ 2}$ and $TRPV3_{2\text{-}APB\ 3}$). **e,** FSC curves calculated between the half maps (black line), atomic model and the final map (orange line), and between the model and each half-map (green and blue lines), of each reconstruction.

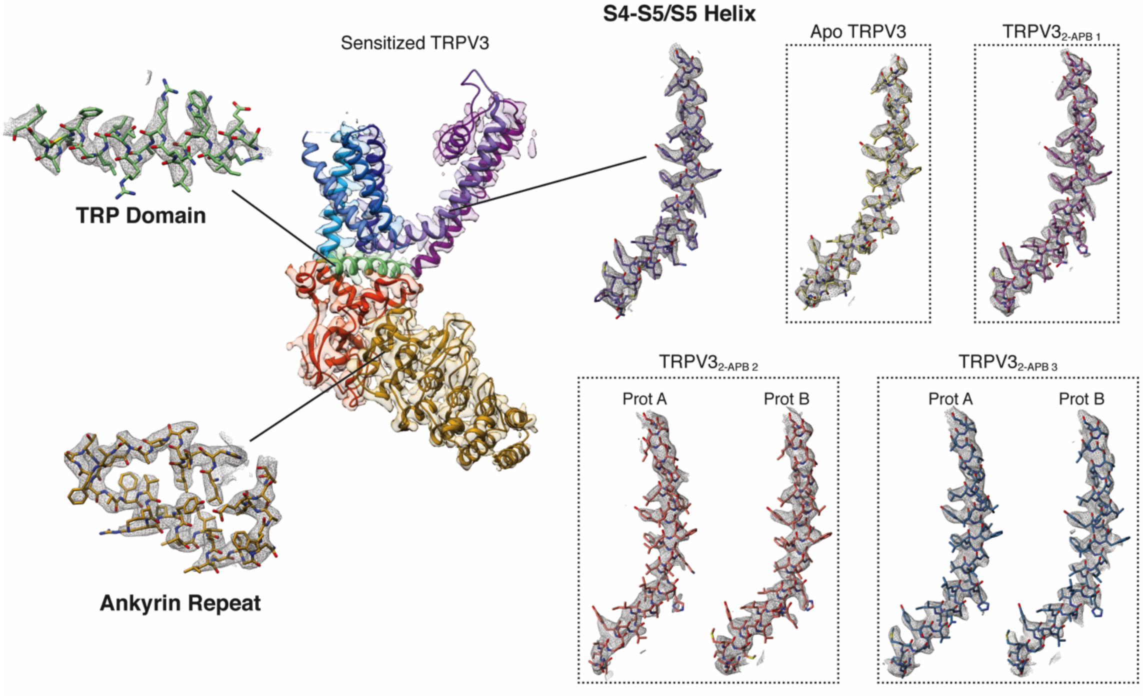

Supplementary Fig. 4 Cryo-EM map quality Cryo-EM density coverage of a single protomer of C4-symmetric sensitized TRPV3 is shown in the central panel, colored according to Fig. 1B. Structural elements of the TRP domain, ARD and S4-S5 linker and S5 helix are indicated. Densities for the S4-S5 linkers and S5 helices of discussed TRPV3 states are shown on the right, and correspond to: C4-symmetric apo TRPV3 (gold) and $TRPV3_{2\text{-APB }1}$ (magenta), top panels; protomers A and B from C2-symmetric $TRPV3_{2\text{-APB }2}$ (pink) and $TRPV3_{2\text{-APB }3}$ (purple), bottom panels. The electron density is depicted as a gray mesh, zoned ~ 2 Å around atoms.

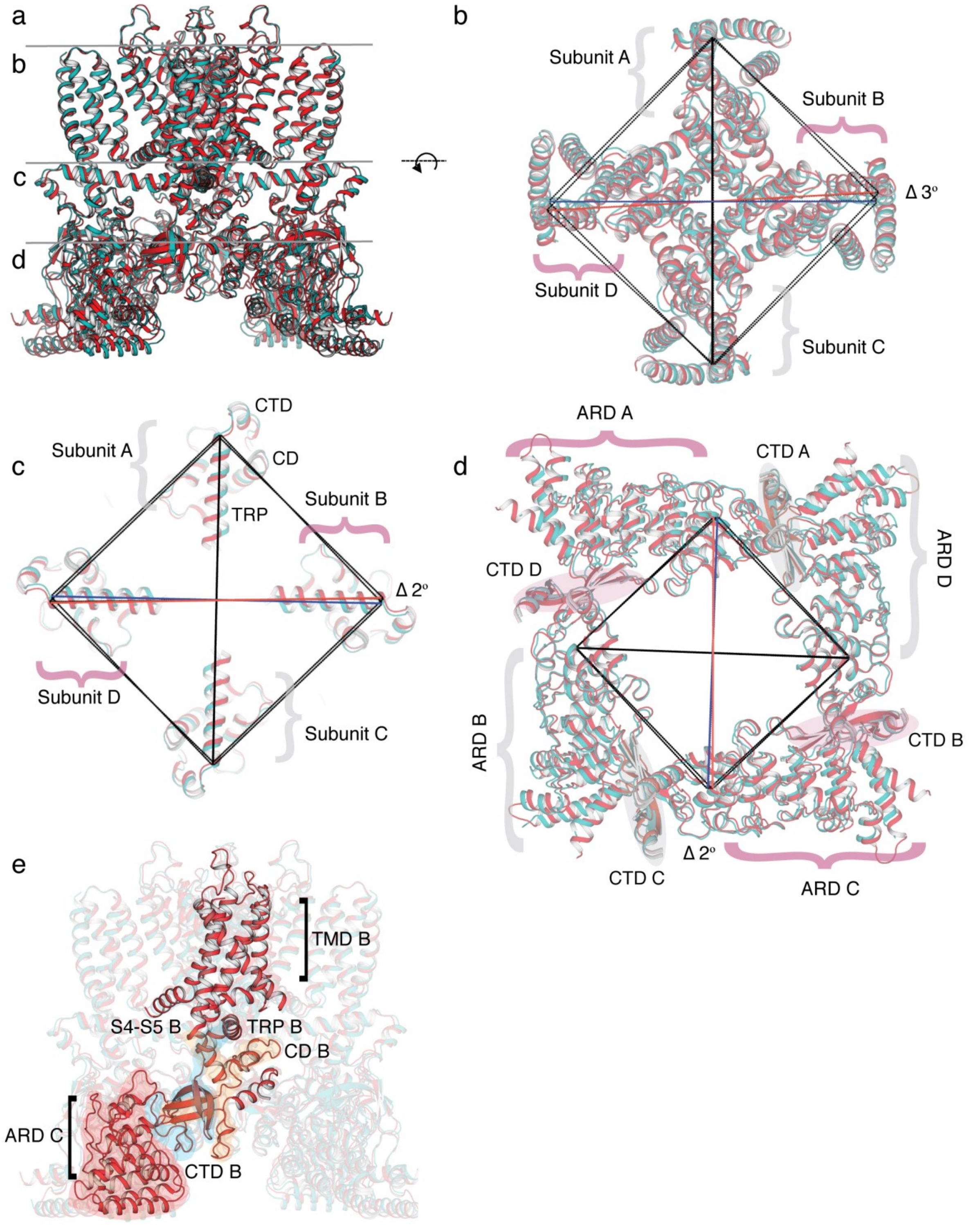

Supplementary Fig. 5 Analysis of motions in TRPV3$_{2\text{-APB }2}$ a, Overlay of sensitized TRPV3 (cyan) and TRPV3$_{2\text{-APB }2}$ (red). Lines represent cross-sections shown in panels **b-d. b,** Top view of the overlay of TRPV3 (cyan) and TRPV3$_{2\text{-APB }2}$ (red). Lines are

drawn between Cα atoms of residues 541. Subunits A and C overlay well, but subunits B and D in $TRPV3_{2\text{-}APB\ 2}$ are rotated by 3° compared to subunits B and D in the sensitized TRPV3. **c,** Top view of the TRP domain, coupling domain (CD) and the proximal CTD. Lines are drawn between the Cα atoms of residues 707. Subunits A and C overlay well, but subunits B and D in $TRPV3_{2\text{-}APB\ 2}$ are rotated by 2° compared to subunits B and D in the sensitized TRPV3. **d,** Top view of the ARD and distal CTD. Lines are drawn between the Cα atoms of residues 327. ARDs B and D align well, but the $TRPV3_{2\text{-}APB\ 2}$ ARDs A and C are rotated by 2° compared to the sensitized TRPV3. **e,** Structural elements that undergo rotation; ARD (highlighted in red), CTD (highlighted in blue), CD (highlighted in orange) and TM.

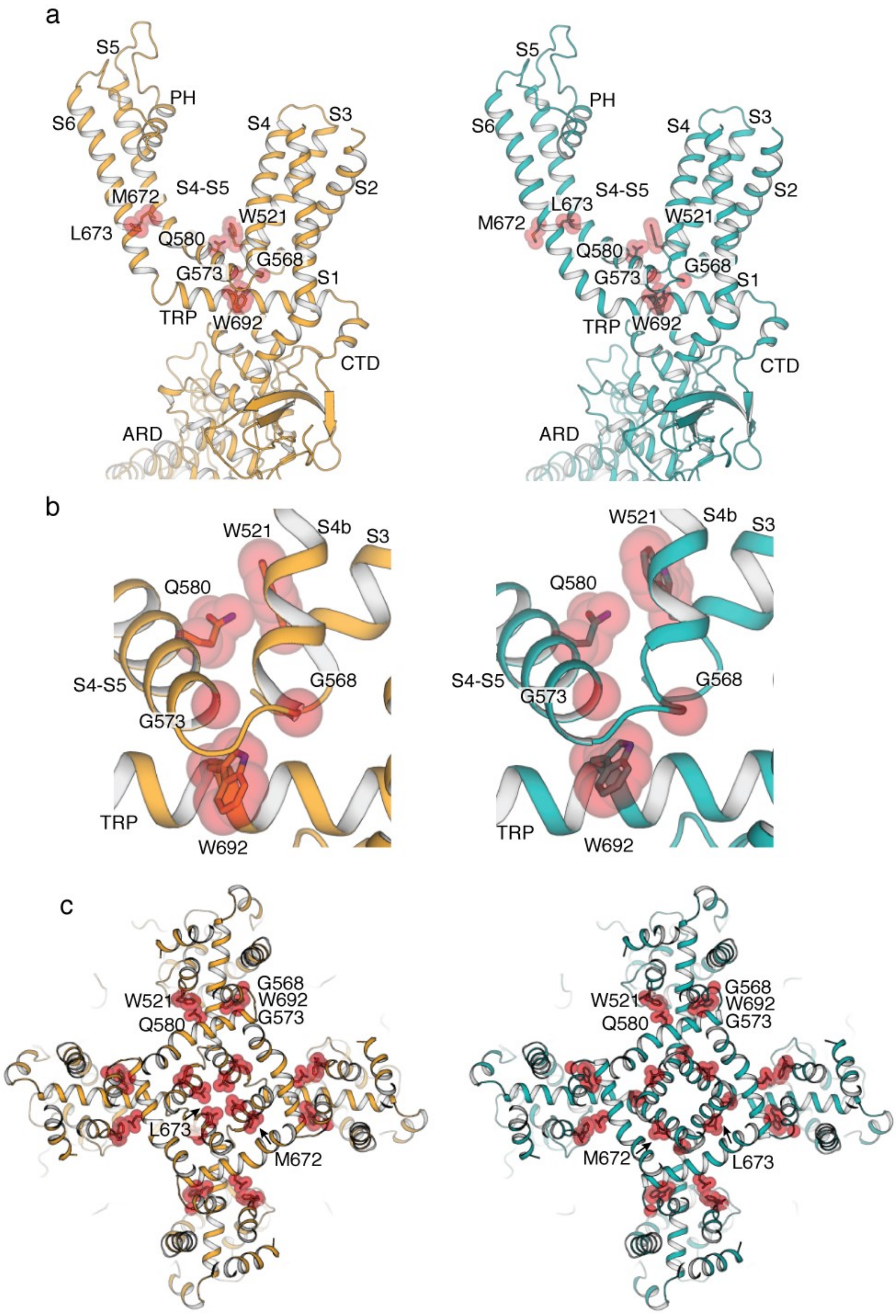

Supplementary Fig. 6 Gain-of-function mutations associated with the Olmsted syndrome Residues W521, G568, G573, Q580, M672, L673 and W692 plotted onto

the structure of apo TRPV3 (gold) and sensitizedTRPV3 (cyan). **a,** Side view of a single protomer showing residues associated with Olmsted syndrome in stick and sphere representation. **b,** Close-up view of the cluster of mutations at the junction of the S4-S5 linker, S4b, S3 and the TRP domain. **c,** Top down view of the channel with the upper part of TM helices removed for ease of viewing. The mutations are shown in stick and sphere representation. Residues M672 and L673 assume different positions depending on the functional state of the channel (apo vs sensitized).

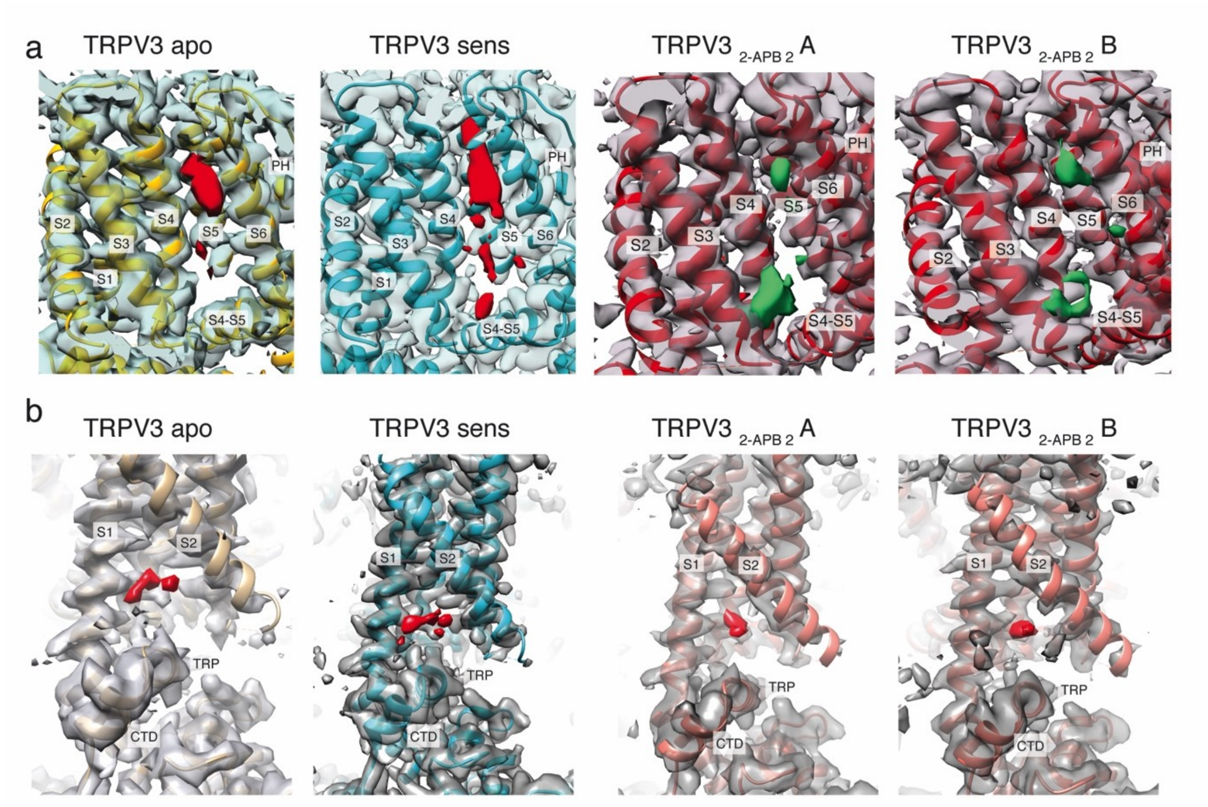

Supplementary Fig. 7 Unassigned densities in the vanilloid binding pocket and the VSLD cavity a, Unassigned density (highlighted in red) in the vanilloid binding pocket in apo TRPV3 (gold), sensitized TRPV3 (cyan) and $TRPV3_{2\text{-}APB\ 2}$ subunits A and B (red) (unassigned density highlighted in green). **b,** Unassigned density in the VSLD cavity (highlighted in red) in the apo TRPV3 (gold), sensitized TRPV3 (cyan) and $TRPV3_{2\text{-}APB\ 2}$ subunits A and B (red).

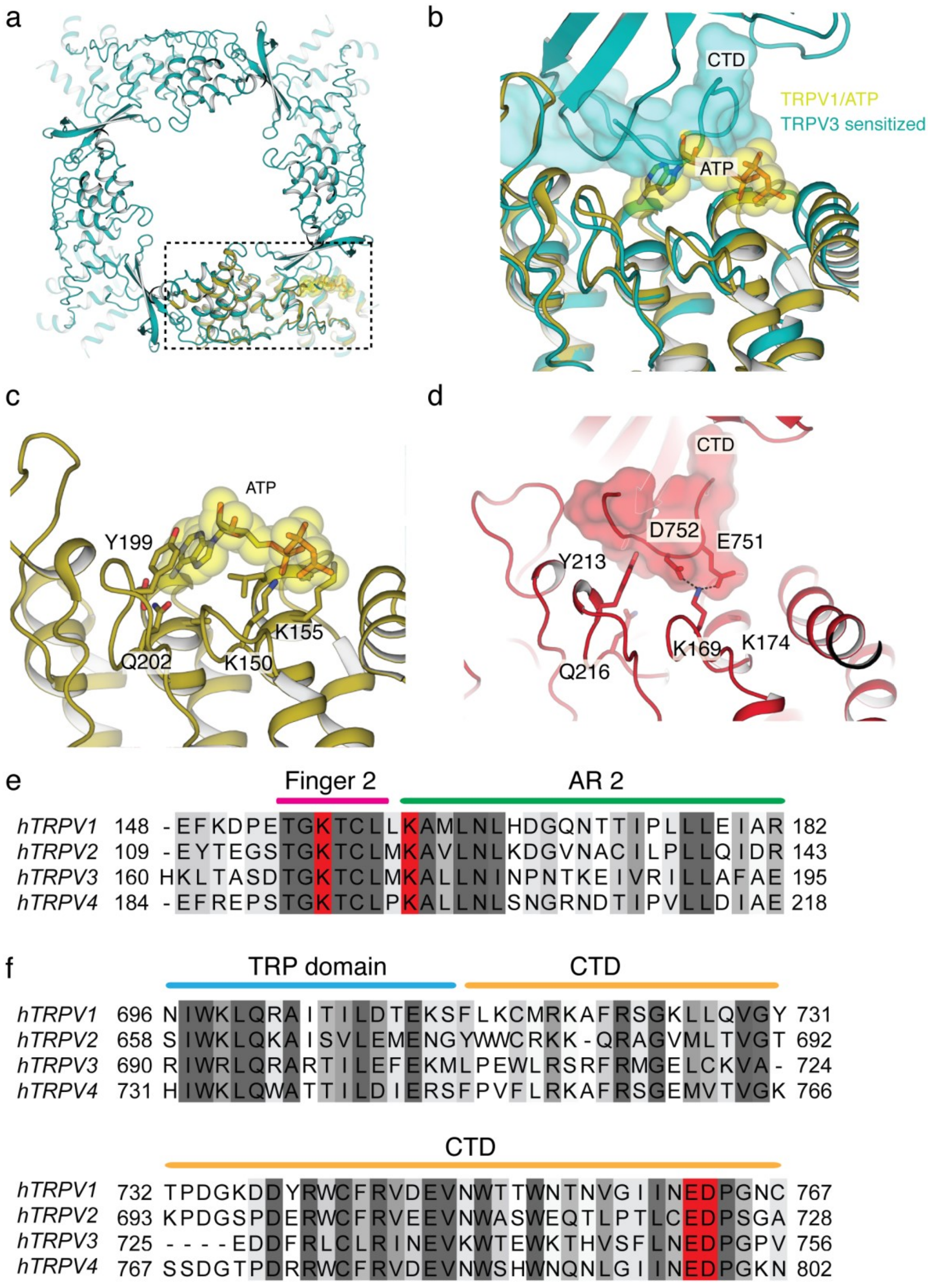

Supplementary Fig. 8 Interactions between CTD and ARD a, Top view of the sensitized TRPV3 ARD (cyan) overlaid with the TRPV1 ARD in complex with ATP (PDB 2PNN) (yellow). ATP is shown in yellow stick and sphere representation. **b,** Close-up of the sensitized TRPV3 ARD (cyan) and TRPV1 ARD/ATP overlay. ATP is shown in yellow stick

and sphere representation. The TRPV3 CTD is shown in surface representation. The position of the extended CTD conincides with the ATP binding site. **c,** Close up of ATP binding in TRPV1 ARD in complext with ATP. ATP is shown in yellow stick and sphere representation. Residues thought to be critical for binding of the ATP molecule, K150, K155, Y199 and Q202 are shown in stick representation. **d,** Close up of the interface formed by the ARD and the extended CTD of a neighbouring protomer in the structure of $TRPV3_{2\text{-}APB\ 2.}$ The extended CTD is shown in surface representation, with acidic residues E751 and D752 shown in stick representation. The ARD residues K169, K174, Y213 and Q216 are shown in stick representation. Dashes indicate interactions between E751 and K169, and D752 and K169. The side chain o K174 was not built due to lack of density. **e**, Sequence alignment of the ARD finger 2 and ankyrin repeat 2 (AR 2) in thermoTRP. Residues K169 and K174 in TRPV3 are conserved (marked in red). **f**, Sequence alignment of the thermoTRP CTD. Conserved acidic residues (E751 and D752 in TRPV3) in the distal CTD are marked in red.

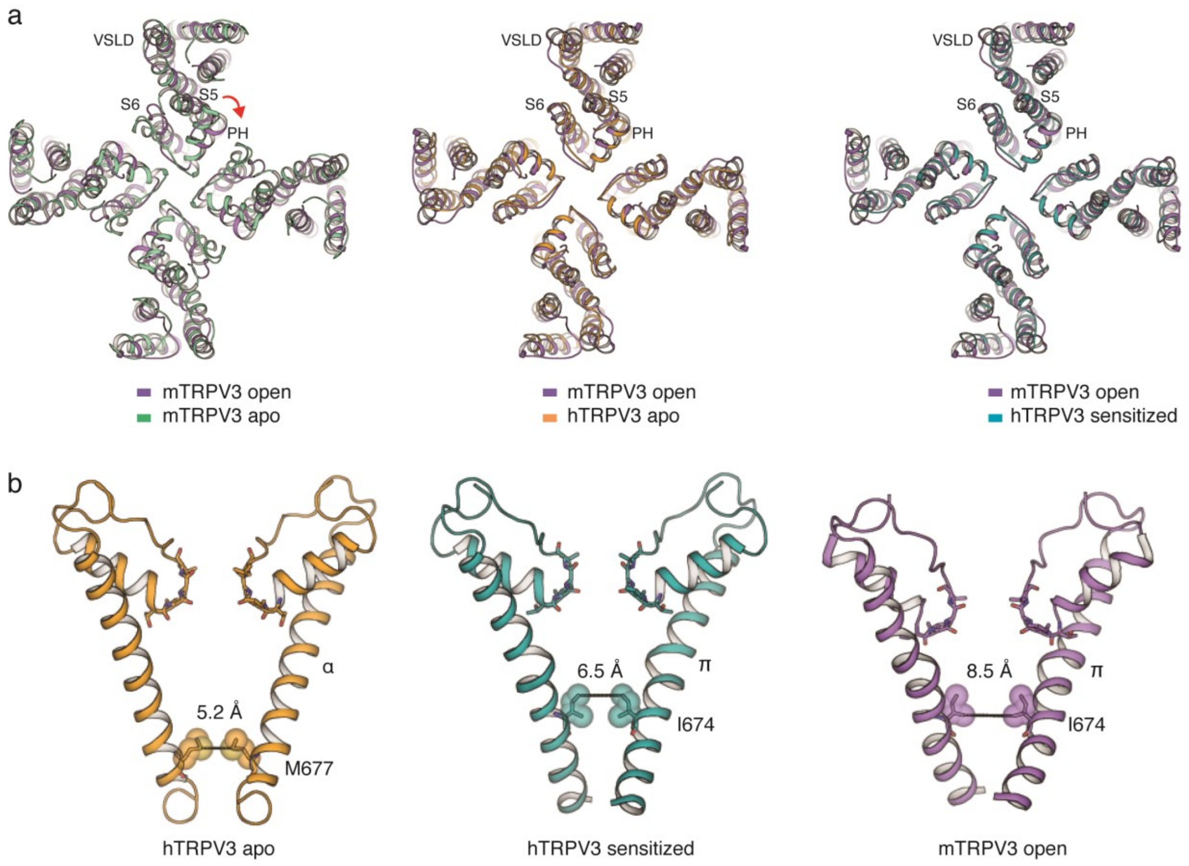

Supplementary Fig. 9 Comaprison of the apo and putative sensitized human TRPV3 channels with the apo and open structures of mouse TRPV3 (PDB ID 6DVW and 6DVZ) a, The pore domain (S5, S6 and PH) of the apo mTRPV3 (green) is rotated compared to the open mTRPV3 structure (magenta). The rotation is not observed in the hTRPV3 apo (gold) or sensitized (teal) structures, in which the pore domain overlays well with that of the open mTRPV3 structure. **b**, The pores of hTRPV3 apo (gold), sensitized (teal) and mTRPV3 open (magenta). The sensitized hTRPV3 structure is closed, despite the presence of a π-helix in S6. The transition to open requires a further bend of the C-terminal part of S6 at the π-helix hinge.

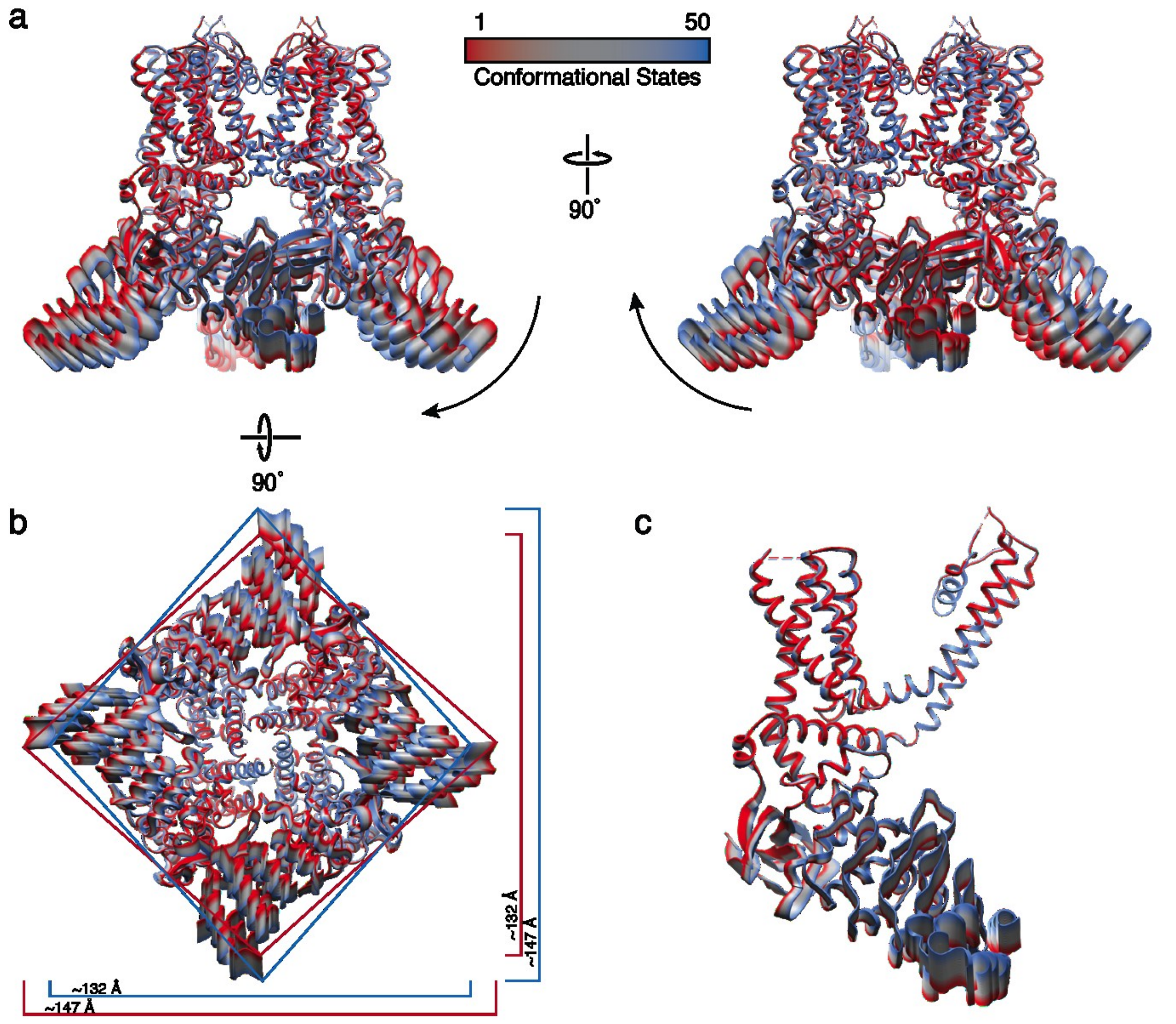

Supplementary Fig. 10 Four-fold to two-fold change in symmetry revealed by normal mode analysis of the apo TRPV3 structure a, Superposition of the 50 conformational states obtained from normal mode analysis of the apo TRPV3 structure. The two-fold symmetric end states are colored red and blue and the four-fold symmetric state is colored gray. **b,** Bottom-up view showing changes in overall dimensions upon deviation from four-fold symmetry. Measurements are taken from opposing ARDs. **c,** A single protomer from each of the 50 conformational states showing most of the movements are localized to the ARD and CTD.

Supplementary Table 1 Primers used for generating point mutations in human TRPV3

Mutation	Forward Primer	Reverse Primer
T96A	gatgtgacagaggccccatccaat	attggatggggcctctgtcacatc
R123G	cggctgaagaagggcatctttgca	tgcaaagatgcccttcttcagccg
E134K	gagggctgcgtgaaggagttggta	taccaactccttcacgcagccctc
M174V	gaagacctgcctggtgaaggcctt	aaggccttcaccaggcaggtcttc
E208V	ttcatcaacgccgtgtacacagag	ctctgtgtacacggcgttgatgaa
T210M	gccgagtacatggaggaggcctat	ataggcctcctccatgtactcggc
D230V	cggcggcagggggtcatcgcagcc	ggctgcgatgaccccctgccgccg
N252S	ggggccttcttcagccccaagtac	gtacttggggctgaagaaggcccc
E264K	ttctacttcggtaagacgcccctg	caggggcgtcttaccgaagtagaa
R293A	gacatcacctcggcggactcacga	tcgtgagtccgccgaggtgatgtc
S363G	aagtacatcctcggtcgtgagatc	gatctcacgaccgaggatgtactt
T394S	ctctacgacctctccaacgtggac	gtccacgttggagaggtcgtagag
T400S	gtggacaccacctcggacaactca	tgagttgtccgaggtggtgtccac
S499D	gccatgtgcatcgatgtgaaagag	ctctttcacatcgatgcacatggc
K501R	tgcatctctgtgagagagggcatt	aatgccctctctcacagagatgca
S516T	tcggatctgcagaccatcctctcg	cgagaggatggtctgcagatccga
W522G	ctctcggatgccgggttccacttt	aaagtggaacccggcatccgagag
S537T	cttgtgatactgactgtcttcttg	caagaagacagtcagtatcacaag
F593S	aagttcttgtctgtatatatcgtg	cacgatatatacagacaagaactt
V594A	ttcttgtttgcatatatcgtgttt	aaacacgatatatgcaaacaagaa
K615E	gagaagtgtcccgaagacaacaag	cttgttgtcttcgggacacttctc
N617E	tgtcccaaagacgagaaggactgc	gcagtccttctcgtctttgggaca
K618Q	cccaaagacaaccaggactgcagc	gctgcagtcctggttgtctttggg
S621G	gacaacaaggactgcggatcctacggca	tgccgtaggatccgcagtccttgttgtc
N648S	aacatccagcagtcctccaagtat	atacttggaggactgctggatgtt
F655I	tatcccattctcattctgttcctg	caggaacagaatgagaatgggata
L677Q	atgctcattgctcagatgggcgag	ctcgcccatctgagcaatgagcat
E726V	tgcaaagtggccgtggatgatttc	gaaatcatccacggccactttgca
V757T	gacccggggcctacaagacgaaca	tgttcgtcttgtaggccccgggtc
